# Critical weather limits for paddy rice under diverse ecosystems of India

**DOI:** 10.3389/fpls.2023.1226064

**Published:** 2023-08-09

**Authors:** Santanu Kumar Bal, Abdus Sattar, Nidhi, Malamal Alickal Sarath Chandran, Abburi Venkata M. Subba Rao, Narayanan Manikandan, Saon Banerjee, Jawahar L. Choudhary, Vijay G. More, Chandra B. Singh, Sandeep S. Sandhu, Vinod Kumar Singh

**Affiliations:** ^1^ All India Coordinated Research Project on Agrometeorology, Indian Council of Agricultural Research (ICAR)-Central Research Institute for Dryland Agriculture, Santoshnagar, Hyderabad, India; ^2^ Dr. Rajendra Prasad Central Agricultural University, Pusa, Samastipur, Bihar, India; ^3^ Department of Agricultural Meteorology and Physics, Bidhan Chandra Krishi Viswavidyalaya (BCKV), Mohanpur, West Bengal, India; ^4^ Department of Agrometeorology, Indira Gandhi Krishi Vishwavidyalaya (IGKV), Raipur, Chhattisgarh, India; ^5^ Department of Agronomy, Dr. Balasaheb Sawant Konkan Krishi Vidyapeeth, Dapoli, Maharashtra, India; ^6^ Department of Agronomy, Chandrashekhar Azad University of Agriculture and Technology, Kanpur, Uttar Pradesh, India; ^7^ Department of Agricultural Meteorology and Climate Change, Punjab Agricultural University (PAU), Ludhiana, Punjab, India

**Keywords:** wet season rice, yield, weather thresholds, phenology, agro-ecological zone

## Abstract

Rice yields are largely influenced by variability in weather. Here, we demonstrate the effect of weather variables viz., maximum and minimum temperatures, rainfall, morning and evening relative humidity, bright sunshine hours on the yield of rice cv. Swarna, grown across five rice ecologies of India through field experiments during *kharif* (wet) season (Jun-Sept.). Critical thresholds of weather elements were identified for achieving above average, average and below average yield for each ecology. The investigation could determine how different weather elements individually and collectively affect rice yield in different rice ecosystems of India. While a sudden increase in minimum temperature by 8-10 °C (> 30 °C) during reproductive period resulted in 40-50 per cent yield reduction at Mohanpur, a sudden decrease (< 20 °C) caused yield decline at Dapoli. The higher yields may be attributed to a significant difference in bright sunshine hours between reproductive phases of above-average and below-average yield years (ranging from 2.8 to 7.8 hours during P5 stages and 1.7 to 5.1 during P4 stages). Rice cultivar Swarna performed differently at various sowing dates in a location as well as across locations (6650 kg ha^-1^ at Dapoli to 1101 kg ha^-1^ at Samastipur). It was also found that across all locations, the above average yield could be associated with higher range of maximum temperature compared to that of below average yield. Principal component analysis explained 77 per cent of cumulative variance among the variables at first growth stage, whereas 70 per cent at second growth stage followed by 74 per cent and 66 per cent at subsequent growth stages. We found that coastal locations, in contrast to inland ones, could maximize the yield potential of the cultivar Swarna, due to the longer duration of days between panicle initiation to physiological maturity. We anticipate that the location-specific thresholds of weather factors will encourage rice production techniques that are climate resilient.

## 1 Introduction

Rice is the third most important cereal crop in the world after maize, wheat and is widely grown across 115 countries with a total production of 517.60 million tonnes (FAO, 2023). It is estimated that about 90 per cent of rice production is achieved in Asia, where about 60 per cent of the world population lives (Pazhanisamy et al., 2020). About 3 billion people around the world consume rice as staple food (Jayapriya et al., 2016). In India, about 43 per cent of total food grain production comes from rice and constitutes about 46 per cent of total cereal production of the country. India contributes about 26.7 and 23.5 per cent of total global rice area and production, respectively.

Being a widely adapted plant, rice is cultivated in wide range of ecosystems i.e. from upland to highly submerged areas. Most of the rice in India is grown under rainfed condition during wet season (June-September) with the receipt of monsoon rainfall. The quantum and distribution of monsoon rainfall, which is the major source of water for rice cultivation, has become erratic during recent years due to climate variability (Ishfaq et al., 2020; Sattar and Srivastava, 2020). The productivity of the crop depends on a wide range of factors viz. land situations, cultivars, weather, planting window and management practices. One of the major constraints of rice production in India is related to climate (temperature, rainfall and solar radiation) variability in the recent years (Pathak et al., 2018). Under such situation, the optimum weather requirement for achieving higher yields need to be quantified. This will help in developing management options for achieving higher rice productivity in the country. Sanchez et al. (2014) opined that optimum temperature for vegetative growth of rice is about 28 °C and optimum temperature for grain filling is about 21.7–26.7 °C. Ahmed et al. (2015) observed that 1000-grain weight and seed-setting rate decreased beyond temperature of 27.0 °C. Nian-bing et al. (2021) studied the effect of solar radiation and temperature on rice in lower reaches of the Huai river basin, China and found temperature being the main limiting factor in realizing higher yields.

Change in rainfall pattern, variability in temperature and duration of bright sunshine hours during crop growing season (monsoon/*kharif* season) affect rice production. The yield response of rice depends on transplanting dates and prevailing weather conditions at different growth stages (Sattar et al., 2017; Sattar and Srivastava, 2020) besides the influence of biotic factors such as insect-pests and diseases. The effect of weather on crop growth and yield could be quantified by collecting data on phenology, growth and yield attributes by conducting multi-year/location field experiments.

Several authors have tried to establish crop-weather relationship at a regional scale in India. However, information on critical weather thresholds on rice particularly long-duration one is non-existing at pan- India level. In the absence of such critical inputs, policymakers at the national level find it hard on many occasions to assess the impact of weather-induced variability and to take policy decisions on rice production under stress situations. Accordingly, a coordinated field experiment, covering varied agro-ecological zones of India, has been conducted focusing on the impact of weather on rice growth and production. The present study will definitely fill the existing vacuum by generating critical weather-based thresholds for achieving higher yields of rice and thus, helping the farming community to mitigate climatic risks based on these critical weather limits developed different phenophases.

**Figure 1:**
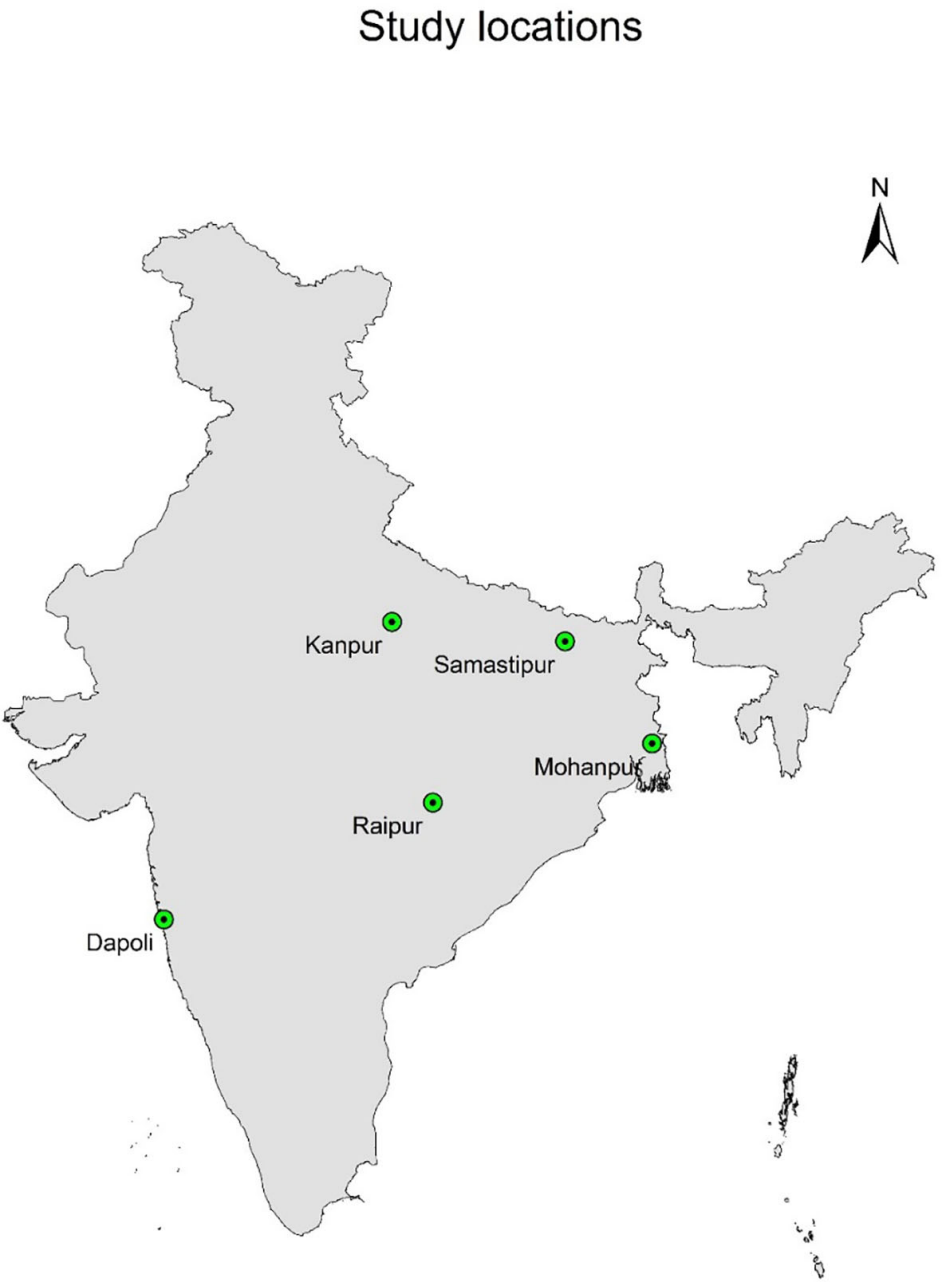
Study areas (major rice growing agro-ecologies of India).

Keeping in mind the above facts, we selected five prominent rice growing ecologies spread across India (**Figure 1**). The response of rice growth and yield were evaluated using *Swarna* variety of rice grown in different sowing windows in these regions with the objectives; quantifying response of rice yield to weather parameters in all these prominent selected rice ecologies of India; characterizing optimum range of weather parameters for achieving higher yields; assessing the contribution of weather variables at different growth phases across rice growing ecologies.

## 2 Materials and methods

### 2.1 Experimental details

Field experiments were conducted at five locations viz. Samastipur, Mohanpur (East India); Kanpur (North India); Raipur (Central India) and Dapoli (West India) representing various agro-ecologies spread across India (**Figure 1**). The study was conducted under All India Coordinated Research Project on Agrometeorology (AICRPAM) funded by Indian Council of Agricultural Research, Ministry of Agriculture and Farmers’ Welfare (MoAFW), Govt. of India for a period of four years (2013 to 2017) at Samastipur, Mohanpur and Dapoli. At Raipur and Kanpur, the experiments were conducted during *kharif* seasons of 2013 to 2016. The details of field experiments, agroclimatic conditions and coordinates of different locations are given in **Table 1**. Long duration (150 days) rice cultivar *Swarna* was grown during wet season (*kharif* season, June-September) at all these locations. The crop was raised with recommended dose of fertilizers and other management practices of the respective regions (**Table 1**).

**Table 1:** Geographic, soil, climate and experimental details of study locations.

Particulars		Locations				
		Samastipur	Mohanpur	Kanpur	Raipur	Dapoli
Latitude		25 °58’N	22 °58’ N	26^0^ 30’N	21°14’ N	17°45’N
Longitude		85 °40’E	88 °31’ E	80^0^ 19’ E	81°39’E	73°26’ E
Altitude (m)		52	9.7	125.9	298	250
Soil texture		Sandy loam	Sandy loam	Sandy loam	Clay loam	Clay loam
Tmax (°C)		33.2	35.0	34.4	32.1	28.9
Tmin (°C)		25.5	25.5	26.0	23.0	22.9
Rainfall (mm)		1028.0	780.0	679.5	1044.8	3384.10
Experimental period		2013-2017	2013-2017	2013-2016	2013-2016	2013-2017
Fertilizers rate (N:P_2_O_5_:K_2_O) kg ha^-1^		120:60:40	80:60:60	120:60:60	100:60:40	100-50-50
Fertilizer schedule		50 per cent of N and full amount of P_2_O_5_ and K_2_O as basal. Remaining N in two equal splits at tillering and PI stage	50 per cent of N and total amount of P_2_O_5_ and K_2_O @ last ploughing and 25 per cent N @ 21 days after DAT and 25 per cent @ 45 DAT	50 per cent of N and full amount of P_2_O_5_ and K_2_O as basal. Remaining ½ N as top dressed in two equal splits at the tillering 58 DAS and PI stage (106 DAT)	50 per cent of N and full amount of P_2_O_5_ and K_2_O @ transplanting, 1/4 N -20 DAT and 1/4 N- 40 DAT	40 per cent of N and full amount of P_2_O_5_ and K_2_O per cent@ transplanting 40 per centN@30 DAT and 20 per cent N @60 DAT
Weeding		Two hand weedings, One at 30 DAT and another at 60 DAT were done	Two hand weedings. One at 21 DAT and another at 45 DAT were done	Two hand weedings. First before tillering and second in PI stage	20-25 DAT (as per field situation) and after that as per need basis 1-2 times	One hand weeding @ 60 DAT

DAT, Days after transplanting; PI, Panicle initiation stage.

The experiments were laid out in split plot design with three varieties. However, in the present study, we have considered only one variety Swarna as it was the only common variety across the five locations. At Samastipur, the crop was raised on four sowing dates viz. 31 May, 15 June, 30 June and 15 July encompassing dates from early to late planting for exposing the crop to different growing environments.

At Mohanpur, the crop was sown on four dates *viz.* 31 May, 10 June, 25 June and 10 July; whereas at Kanpur (15 June, 25 June and 5 July) and Raipur (1 June, 15 June and 30 June) and Dapoli (7 June, 17 June and 27 June), three dates of sowing were followed.

At each location, 21-25-day old seedlings were used for transplanting. The crop was raised with receipt of monsoon rainfall and supplemental irrigations were provided to the crop to ensure that moisture level remains at saturation.

### 2.2 Climate and soils of the study area

**Figure 2:**
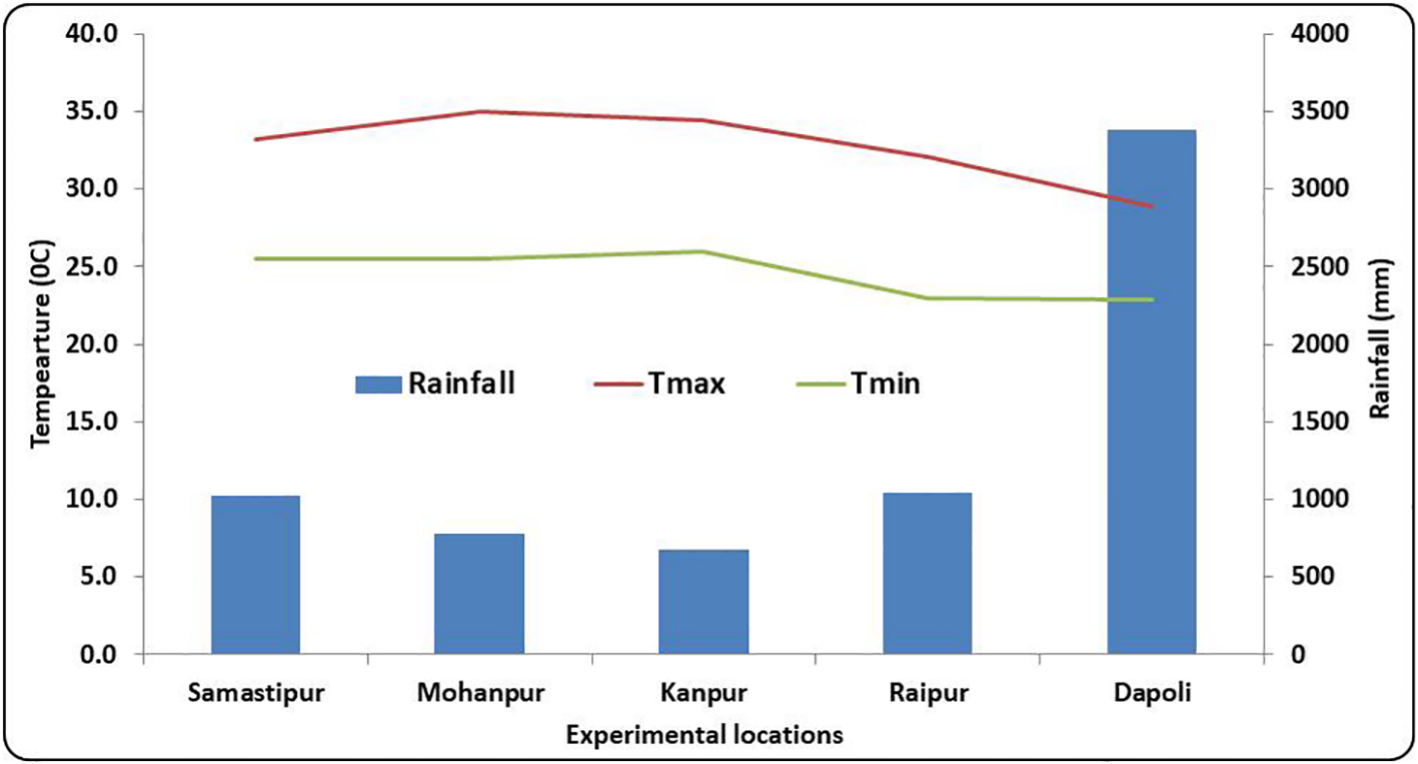
Spatial variation in rainfall, maximum temperature (T_max_), minimum temperature (T_min_) during rice growing season across five locations.

The experimental sites at Mohanpur, Samastipur and Kanpur are situated in lower, middle and upper Gangetic plains of India, respectively. Raipur was selected from central India, while Dapoli represents Konkan region of Western Ghats in South-Western India. The altitude of the locations ranges from 9.7 m AMSL at Mohanpur to 298 m at Raipur. A wider variability exists among all the locations with respect to rainfall amount, rainfall distribution and temperature (**Figure 2**). Considering the minimum temperature, almost similar pattern is observed at Kanpur, Samastipur and Mohanpur, all of which are located in the Indo-Gangetic plains. However, Dapoli experiences lowest temperature during rice growing season. In case of minimum temperature, lowest value was observed at Dapoli region, while Indo-Gangetic plains experienced higher values. Dapoli experiences very high rainfall during *kharif* season, owing to its location in Western Ghat ranges near Arabian sea, while Kanpur receives the lowest among all locations. The soils across the study areas vary from sandy loam to clay loam. Soil texture of locations in Indo-Gangetic plain (Samastipur, Kanpur and Mohanpur) is sandy-loam, whereas Dapoli and Raipur have clay-loam textured soil.

### 2.3 Weather data

Weather data on all important parameters viz. maximum temperature, minimum temperature, rainfall, bright sunshine hours (BSH), morning relative humidity and evening relative humidity were collected from agrometeorological observatories located near the experimental sites of each location. All weather parameters from sowing to physiological maturity were collected on daily basis.

### 2.4 Phenological observations

Dates of occurrence of important phenological events of rice crop viz. germination, tillering, panicle initiation, flowering, milk and maturity were recorded for the rice crop at selected locations in all the years. Grain yield and dry weight were recorded at harvest from net plot area from all experimental sites. After the removal of excess moisture from the grains of each plot, the grain yield (kg per plot) was recorded using open pan electronic balance, which was later converted to kg ha^-1^.

### 2.5 Statistical measures to quantify weather effects on growth and yield

Mean values of weather parameters (maximum temperature, minimum temperature, rainfall, sunshine hours, morning relative humidity, evening relative humidity) during different phenophases [transplanting to tillering (P1), tillering to panicle initiations (P2), panicle initiation to flowering (P3), flowering to milk (P4) and milk to physiological maturity (P5)] were worked out. Three critical yield levels were identified for each location viz. above average (M+σ), average (M) and below average yields (M– σ) (Vijaya Kumar et al., 2015). Further, phenophase-wise thresholds of individual weather variables responsible for above average (high), average (optimum) and below average (low) yields were identified. Regression models were fitted between yield and temperature, rainfall, BSS hour, morning and evening relative humidity averaged over different phenological stages. It was used for identifying critical phenological stages and quantifying the effect of weather parameters on yields. Statistical significance of the developed regression models was done using F-test and t-test.

### 2.6 Principal component analysis

PCA is a technique for reducing the dimensionality of large datasets thereby increasing interpretability with minimum information loss. It creates new uncorrelated variables that successively maximize variance. PCA does feature extraction by taking projections of data along axes of maximum variance (principal components) which are independent of one another (Jolliffe, 1986). The PCA as an exploratory data analysis tool involves a dataset with observations on *p* numerical variables, for each of *n* entities or individuals. These data values define *p n*-dimensional vectors x_1_,…,x*_p_* or, equivalently, an *n*×*p* data matrix X, whose *j*th column is the vector **x** *_j_* of observations on the *j*th variable. A linear combination of the columns of matrix X with maximum variance is sought. The linear combinations are given by $\overset{\;}{\underset{\sum }{\;}}\overset{p}{\underset{j=1}{\;}}a_{j}x_{j}=Xa$ , where *a* is a vector of constants $a_{1,}a_{2}\ldots a_{p}$ . The variance of any such linear combination is given by Var(Xa)=a'Sa , where *S* is the sample covariance matrix associated with the dataset and ′ denotes transpose. Hence, identifying the linear combination with maximum variance is equivalent to obtaining a *p*-dimensional vector *a* which maximizes the quadratic form ‘*Sa*’.

In this study, it was used to assess the phase-wise contribution of weather variables towards rice yields at different locations. For each of the five growth stages (phases), principal component analysis (PCA) using the correlation matrix was performed on yield and all the weather parameters to determine which weather variables played critical role in explaining the variation in rice yield at different agroecologies. The principal components with eigen value greater than one was selected for ordination analysis. The first two principal components (PC1 and PC2) were observed to be fulfilling the criteria at each growth stage. The analysis was carried out in R studio software.

## 3 Results

### 3.1 Weather and rice growth across rice ecosystems

The average values of maximum temperature, minimum temperature, rainfall, BSH, morning relative humidity and evening relative humidity prevailed during sowing – physiological maturity (P1 to P5) stages of rice (**Table 2**) indicated large variations during different phenophases and locations. During P1 stage (sowing to tillering), T_max_ ranged from 28.6 °C at Dapoli to 33.8 °C at Kanpur. On the other hand, the higher T_min_ was recorded at Mohanpur (26.3 °C) and the lower at Dapoli (23.8 °C).

**Table 2:** Weather conditions during different phenological stages of rice at the study locations during *kharif seasons of* 2013-2016.

PS	Weather variables	Locations				
		Eastern India		Northern India	Central India	Western India
		Samastipur	Mohanpur	Kanpur	Raipur	Dapoli
P1	Tmax (°C)	33.0 ( ± 1.4)	33.9 ( ± 1.2)	33.8 ( ± 1.3)	31.8 ( ± 1.4)	28.6 ( ± 0.7)
	Tmin (°C)	25.6 ( ± 0.9)	26.3 ( ± 1.5)	24.9 ( ± 1.0)	25.0 ( ± 0.6)	23.8 ( ± 0.5)
	BSH (hours)	5.5 ( ± 0.9)	4.4 ( ± 1.4)	4.9 ( ± 0.8)	3.4 ( ± 0.7)	2.1 ( ± 1.1)
	Rainfall (mm)	350.0 ( ± 126.6)	211.7 ( ± 153.9)	382.5 ( ± 175.1)	699.3 ( ± 171.2)	2183.9 ( ± 653.2)
	RH-1 (m) (per cent)	91.9 ( ± 6.7)	95.6 ( ± 1.9)	85.6 ( ± 4.6)	77.7 ( ± 19.1)	93.9 ( ± 2.3)
	RH-2 (e) (per cent)	70.0 ( ± 3.3)	82.0 ( ± 5.3)	69.7 ( ± 7.8)	66.7 ( ± 13.9)	88.2 ( ± 5.0)
P2	Tmax (°C)	32.8 ( ± 1.1)	33.0 ( ± 0.7)	33.5 ( ± 1.2)	31.1 ( ± 1.0)	28.4 ( ± 0.6)
	Tmin (°C)	25.0 ( ± 0.9)	26.3 ( ± 0.3)	23.6 ( ± 1.7)	24.8 ( ± 0.8)	23.5 ( ± 0.4)
	BSH (hours)	5.0 ( ± 0.8)	4.3 ( ± 0.7)	6.4 ( ± 0.6)	4.0 ( ± 0.9)	2.9 ( ± 1.5)
	Rainfall (mm)	329.2( ± 162.4)	401.1 ( ± 169.5)	155.2 ( ± 79.6)	213.4 ( ± 110.0)	406.2 ( ± 137.9)
	RH-1 (m) (per cent)	90.0( ± 1.1)	95.8 ( ± 1.0)	85.6 ( ± 2.0)	87.8 ( ± 6.7)	94.4 ( ± 0.9)
	RH-2 (e) (per cent)	69.2 ( ± 4.8)	79.4 ( ± 3.3)	65.8 ( ± 4.7)	75.0 ( ± 6.7)	85.9 ( ± 3.4)
P3	Tmax (°C)	32.3 ( ± 1.0)	33.7 ( ± 0.5)	32.6 ( ± 2.1)	31.9 ( ± 1.2)	29.2 ( ± 0.7)
	Tmin (°C)	23.3 ( ± 1.2)	25.8 ( ± 0.9)	18.9 ( ± 2.3)	24.4 ( ± 0.6)	22.8 ( ± 0.6)
	BSH (hours)	6.3 ( ± 1.1)	5.7 ( ± 1.4)	7.1 ( ± 1.2)	5.8 ( ± 1.5)	4.0 ( ± 0.9)
	Rainfall (mm)	107.6 ( ± 117.1)	151.7 ( ± 91.2)	17.1( ± 24.6)	126.4 ( ± 125.7)	402.5 ( ± 302.7)
	RH-1 (m) (per cent)	90.0 ( ± 1.2)	94.7 ( ± 2.6)	86.5 ( ± 3.0)	91.0 ( ± 5.8)	93.6 ( ± 2.1)
	RH-2 (e) (per cent)	62.7 ( ± 7.2)	73.7 ( ± 4.7)	52.6 ( ± 10.8)	66.7 ( ± 8.1)	83.5 ( ± 3.9)
P4	Tmax (°C)	31.5 ( ± 1.7)	33.5 ( ± 1.6)	31.7 ( ± 2.2)	31.5 ( ± 1.4)	30.9 ( ± 1.6)
	Tmin (°C)	21.1 ( ± 2.2)	24.8 ( ± 1.6)	16.0 ( ± 2.2)	23.5 ( ± 1.6)	22.7 ( ± 0.6)
	BSH (hours)	6.4 ( ± 1.5)	6.3 ( ± 1.4)	7.2 ( ± 1.3)	6.6 ( ± 3.3)	4.6 ( ± 0.5)
	Rainfall (mm)	38.4 ( ± 95.2)	49.5 ( ± 71.2)	1.7 ( ± 5.4)	41.1 ( ± 67.8)	238.5 ( ± 242.6)
	RH-1 (m) (per cent)	89.0 ( ± 1.8)	92.4 ( ± 5.1)	86.5 ( ± 3.5)	90.1( ± 9.6)	93.4 ( ± 2.1)
	RH-2 (e) (per cent)	56.2 ( ± 10.6)	66.4 ( ± 8.8)	45.3 ( ± 7.9)	63.8 ( ± 11.5)	78.4 ( ± 7.7)
P5	Tmax (°C)	29.9 ( ± 1.5)	32.8 ( ± 1.3)	30.3 ( ± 1.1)	31.3 ( ± 1.4)	33.0 ( ± 0.9)
	Tmin (°C)	17.2 ( ± 2.2)	23.8 ( ± 2.4)	13.7 ( ± 1.7)	20.3 ( ± 1.8)	21.0 ( ± 1.8)
	BSH (hours)	6.5 ( ± 0.9)	6.2 ( ± 1.1)	6.1 ( ± 1.2)	7.5 ( ± 1.3)	7.2 ( ± 1.1)
	Rainfall (mm)	22.5 ( ± 56.3)	88.8 ( ± 79.2)	3.1 ( ± 7.3)	17.3 ( ± 21.8)	14.5 ( ± 25.9)
	RH-1 (m) (per cent)	87.0 ( ± 1.1)	91.7 ( ± 4.8)	86.2 ( ± 3.6)	87.6 ( ± 12.5)	78.4 ( ± 7.7)
	RH-2 (e) (per cent)	48.9 ( ± 5.0)	63.1 ( ± 9.2)	44 ( ± 5.9)	47.0 ( ± 8.5)	67.9 ( ± 6.7)

P1: Sowing to Tillering, P2: Tillering to Panicle initiation, P3: Panicle initiation to flowering, P4: Flowering to milk; P5: Milk to Physiological maturity.

During this stage, large variations in rainfall and BSH were observed across the locations. During early growing period up to panicle initiation stage, rice crop experienced higher values of temperature, rainfall and BSH at all locations, while the lowest values experienced at Dapoli, located in hilly areas of Western Ghat. Duration of optimum BSH during flowering to milk stage is a crucial factor for achieving better growth and yield of rice and daily value of BSH in the range of 7 to 8 hours during flowering phase led to enhanced grain yield (Sattar et al., 2017). The study indicated that at all locations except Dapoli, BSH varied from 6.3 to 7.5 hours during flowering to milk stage. On the contrary, rice at Dapoli availed only 4.6 hours BSH during this particular phenophase. The magnitude of BSH increased significantly from P4 to P5 stage at Raipur and Dapoli, facilitating satisfactory growth and higher yield. Whereas, the values of temperature and BSH hours drastically reduced at later stages of rice growth. At Kanpur, flowering to milk stage encountered very low temperature much below 20 °C, which is an important climatic constraint for realizing potential productivity.

Considering the availability of rainfall during critical growth phases of rice i.e., from panicle initiation to milk, the crop tends to face moisture stress at Kanpur, probably due to occurrence of frequent dry spells as a result of early cessation of south-west monsoon. Based on prevalence of temperature and rainfall at later critical growth phases at Kanpur, it could be inferred that late sowing of rice in this region is not conducive for satisfactory growth and yield. Prevalence of variable maximum temperature might have affected proper grain setting.

### 3.2 Modelling impact of weather during critical growth stages on rice productivity

#### 3.2.1 Samastipur (Middle Gangetic Plain)

Linear regression models of yield with weather elements during all phenological phases of rice at study locations were developed (**Supplementary Table 1**). The results revealed that maximum temperature at Samastipur region had a significant influence on grain yield during all phenological phases, except P1 stage, while minimum temperature did not show significant impact on yield during any of the growth stages. Based on the degree of sensitivity to maximum temperature, panicle initiation to flowering stage (P4) and flowering to milk stage (P3) were identified as critical stages for thermal stress owing to higher absolute t values of 4.149 and 3.769, respectively during these stages. Craufurd et al. (2013) also reported that both intensity and duration of thermal stress impact crop productivity. Minimum temperature at Samastipur did not pose any constraint in realizing potential yield provided sowing is not delayed beyond 15 July. With unit increase of minimum temperature during P3 stage, the grain yield was enhanced at the rate of 692 kg ha^-1^. BSH hours had highly significant impact on rice growth and yield at P3, P4 and P5 stages. The highest sensitivity was observed during P3 stage [t-value of 4.61 (p<0.01)]. Since most of the rice in this region is grown under rainfed condition, rainfall during P4 had significant positive impact. The morning relative humidity during P1 stage had positive influence on good growth and seedling establishment. This could be ascribed to the fact that excessive moisture fed by initial burst of monsoon rainfall at initial growth stage of crop. Rice requires a fairly high degree of humidity (80-85 per cent) for shoot growth during initial vegetative phase (Sridevi and Chellamuthu, 2015).

#### 3.2.2 Mohanpur (Lower Gangetic Plain)

T_max_ had significant influence on rice productivity at Mohanpur situated in eastern India. Higher t and lower p values during P4 (flowering to milk), followed by P1 (sowing to tillering) stages indicated the sensitivity of cv. Swarna towards thermal stress. T_min_ and BSH had significant impact during P3 (panicle initiation to flowering) to P5 (milk to physiological maturity) stages on yield, with P4 as the most sensitive stage. During the fag end of rice growing season (October-November), frequent cyclonic activities fed by Bay of Bengal generates cloudy weather and incessant rains for a few days even for a week leading to reduced sunshine hours in this region. In terms of its relevance of significance and t values, rainfall during P1 and P4 growth stages appeared to be greatly impacting rice yield. However, morning relative humidity during P1 stage like that of Samastipur region and evening relative humidity during P5 stage had negative relationship with yield. This may probably be due to late receipt of rainfall from cyclonic activities during physiological maturity to harvest.

#### 3.2.3 Kanpur (Upper Gangetic Plain)

At Kanpur (northern India), thermal sensitivity of the crop to T_max_ and T_min_ was observed during P4 stage. Sunshine hours had significant positive and negative influence on the crop during P3 and P2 stages, respectively. Deng et al. (2009) reported that grain filling and yield were affected by shading. As far as rainfall is concerned, significant positive (P2 and P3) and significant negative (P4 and P5) impact was observed and P4 was the most moisture sensitive stage. Yield of rice was found to be significantly affected by morning relative humidity during P3 and P4 stages, while no effect was observed in case of evening relative humidity.

#### 3.2.4 Raipur

In Raipur (central India), P3 and P4 stages were the most thermal sensitive stages to T_max_ and T_min_, respectively. P2 followed by P3 were identified as most sensitive stages to the availability of BSH. Rainfall had significant impact on grain yield on major growth stages. Unlike other places, relative humidity registered significant impact on all growth stages except P1 stage.

#### 3.2.5 Dapoli

At Dapoli in South-Western India, only T_max_ during P1 stage showed significant positive effect on rice yield. On the other hand, based on probability values associated with t statistics, the significant positive effect of BSH was the highest during P3 stage. Rainfall during P1 followed by P5 was the most sensitive stages. RH-1 at P5 and RH-2 during P1 stages showed significant positive influence on yield. Optimum amount of well-distributed rainfall during rice growing period coupled with reduced aberrations in temperature tend to critically decide rice yield (Athar et al., 2018).

### 3.3 Limits of weather variables for different categories of yield

Thresholds of weather elements were worked out for each phenological stage of rice for obtaining three yield categories [above average, average and below average and presented in **Supplementary Tables 2–7**. Identifying the optimum thresholds of weather variables is vital for assessing extreme weather-related risks and trigger values for weather-based crop insurance (Vijaya Kumar et al., 2015).

#### 3.3.1 Maximum temperature (T_max_)

**Figure 3:**
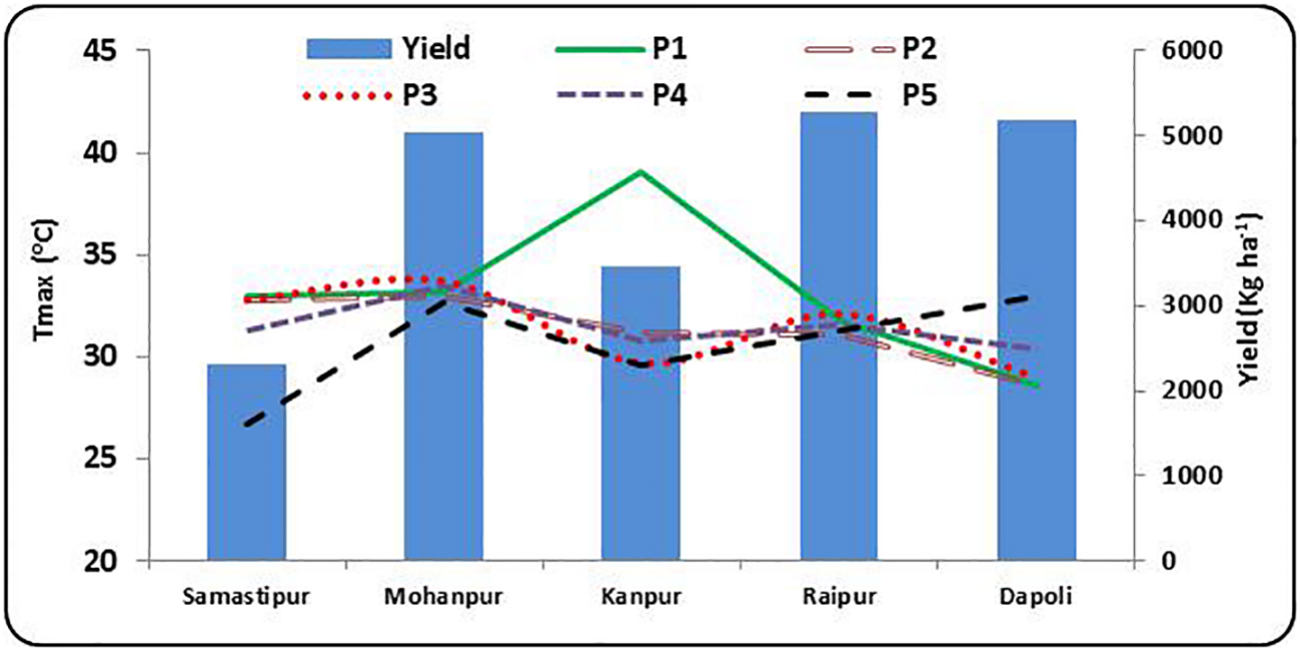
Maximum temperature (T_max_) during different rice growth stages vis-a-vis yield at different rice growing ecologies of India, (P1: Sowing to Tillering, P2: Tillering to Panicle initiation, P3: Panicle initiation to flowering, P4: Flowering to milk; P5: Milk to Physiological maturity).

A gradual decrease in T_max_ with crop growth helped to achieve an average yield (2309 kg ha^-1^) at Samastipur (**Figure 3**; **Supplementary Table 2**). A temperature range of 34.5 to 34.9 °C during P3-P4 stages was found optimum for achieving above average yield (3717 kg ha^-1^). T_max_ greater than 34 °C during P3 and P4, less than 31 °C during P1 and P2 and less than 25 °C during P5 stage contributed for above average yields. At Mohanpur, above average yield was recorded when T_max_ was greater than 36 °C during P3, P4 and P5 stages. Higher temperature (> 35 °C) during P1 and P2 and lower temperature during P3, P4 and P5 (< 32 °C) contributed to below average yield. It is to be noted that, the below average yield of Mohanpur (3459 kg ha^-1^) was on par with the above average yield of Samastipur (3517 kg ha^-1^).

Yield categories of rice recorded at Kanpur (upper Gangetic plains) were in line with maximum temperature for other two locations viz. Samastipur (middle Gangetic plains) and Mohanpur (lower Gangetic plains). Moderate temperature regime (at major phenological stages) at Kanpur compared to Samastipur and Mohanpur perhaps helped in achieving average yield level (3458 kg ha^-1^).

Raipur (central India) achieved average yield (5288 kg ha^-1^) compared to Kanpur (3458 kg ha^-1^) with a difference of more than 1800 kg ha^-1^. However, the range of T_max_ was found almost similar in both the locations especially during P3-P5 stages.

Although average yield of rice (5186 kg ha^-1^) at Dapoli (south-western India) is almost comparable to that of Raipur (5288 kg ha^-1^), the above average rice yield at Dapoli (6650 kg ha^-1^) was higher than Raipur (5991 kg ha^-1^).

#### 3.3.2 Minimum temperature (T_min_)

**Figure 4:**
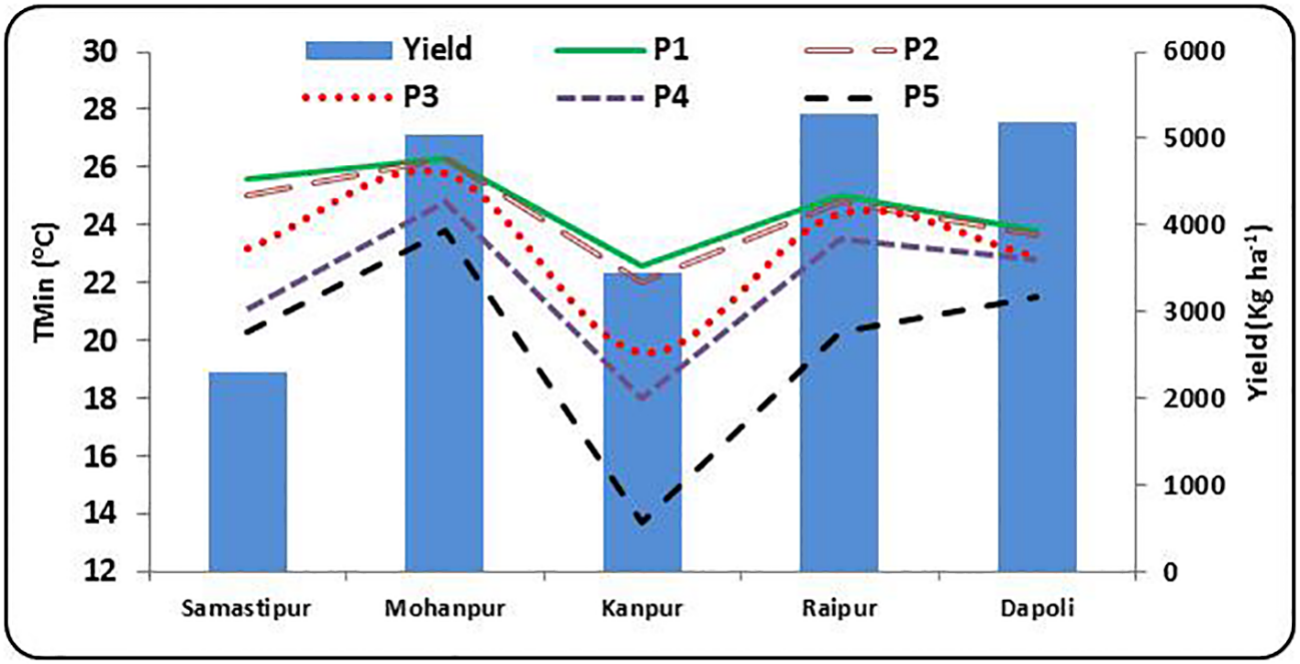
Minimum temperature (T_min_) during different rice growth stages vis-a-vis yield at different rice growing ecologies of India, (P1: Sowing to Tillering, P2: Tillering to Panicle initiation, P3: Panicle initiation to flowering, P4: Flowering to milk; P5: Milk to Physiological maturity).

Like T_max_, the threshold values of T_min_ also showed wide variations across different phonological stages at different locations in India (**Supplementary Table 3**; **Figure 4**).

At Samastipur, the progressive decrease in T_min_ from P2 (29.0 °C) to P5 (19.7 °C) stage favored achieving the above average yield (3517 kg ha^-1^). A higher T_max_ during P1 (>35 °C) and P5 (> 23 °C) might have contributed for the below average yield. A gradual decrease in T_min_ from P1 (25.2 °C) to P5 (19.7 °C) helped in obtaining an above average yield (6624 kg ha^-1^) at Mohanpur. At the same time, a higher T_min_ throughout P2 to P5 stages (above 29 °C) resulted in below average yield (3459 kg ha^-1^). Similar results were obtained for Kanpur, where a consistent reduction in T_min_ from P1 (32.3 °C) to P5 (20 °C) resulted in above average yield (4495 kg ha^-1^). A higher T_min_ during P5 (greater than 22 °C) resulted in below average yield (2421 kg ha^-1^). In Raipur, no significant impact of T_min_ on rice yield was observed. Consistently higher T_min_ (> 32 °C) during P1 to P4 and comparatively lower T_min_ (< 30 °C) contributed for above average yield (6650 kg ha^-1^) at Dapoli. Substantially low T_min_ (< 25 °C) during most of the growth period resulted in below average yield (4463 kg ha^-1^).

#### 3.3.3 Rainfall

**Figure 5:**
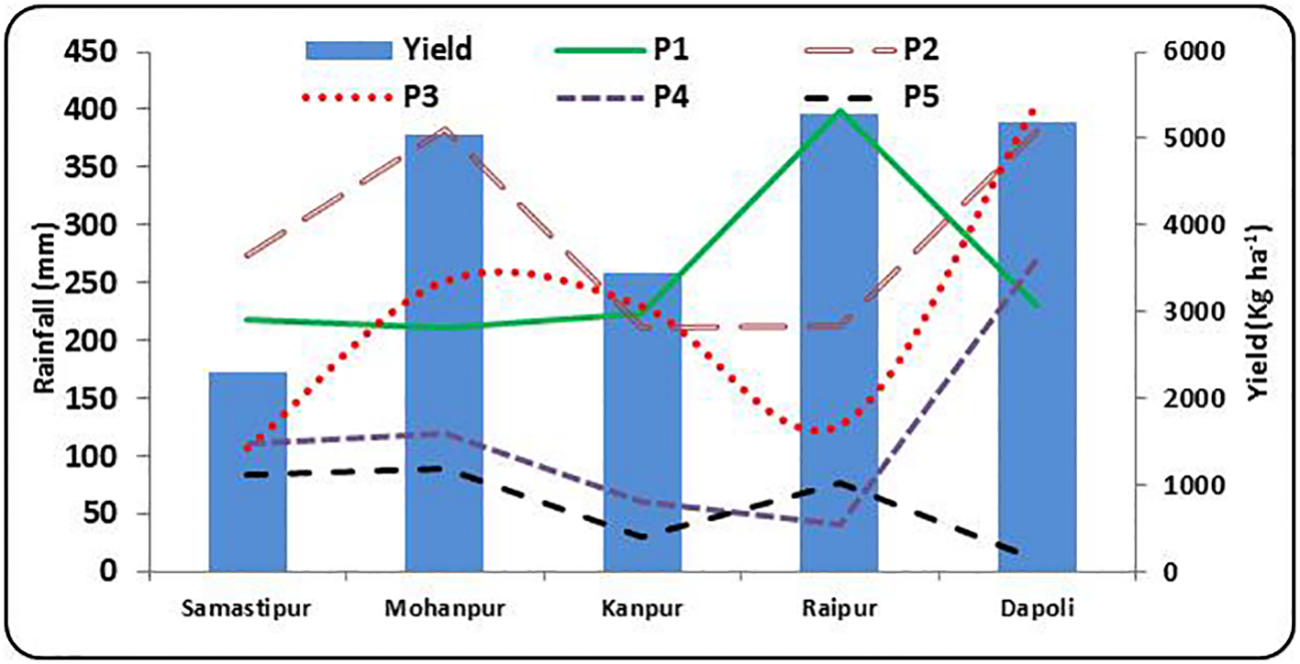
Rainfall during different rice growth stages vis-a-vis yield at different rice growing ecologies of India, (P1: Sowing to Tillering, P2: Tillering to Panicle initiation, P3: Panicle initiation to flowering, P4: Flowering to milk; P5: Milk to Physiological maturity).

The thresholds of rainfall required for different phenological stages for above average, average and below average yields at different locations are presented in **Supplementary Table 4** and **Figure 5** represents values of thresholds rainfall and optimum yield at different locations. A difference of around 1000 mm in total rainfall during crop growing period was observed between above average yield (3517 kg ha^-1^) year and below average yield (1101 kg ha^-1^) at Samastipur. A rainfall of 660.6 mm during panicle initiation to flowering (P3) stage has caused below average yield (3459 kg ha^-1^) at Mohanpur. The above average yield (6624 kg ha^-1^) may be attributed to higher rainfall of 2004 mm, compared to 1587 mm in case of below average yield. Similar result was found at Dapoli also, where a rainfall of 1040 mm during P3 stage resulted in below average yield of 4463 kg ha^-1^, compared to above average yield of 6650 kg ha^-1^. Samui et al. (1998) observed that rice yield in Kerala (India) appeared to remain more or less stationary so long the total rainfall remains within 900 mm and decreases as the total rainfall exceeds 900 mm. They found optimum value of the total rainfall was 900 mm for higher production.

#### 3.3.4 Bright sunshine hours

**Figure 6:**
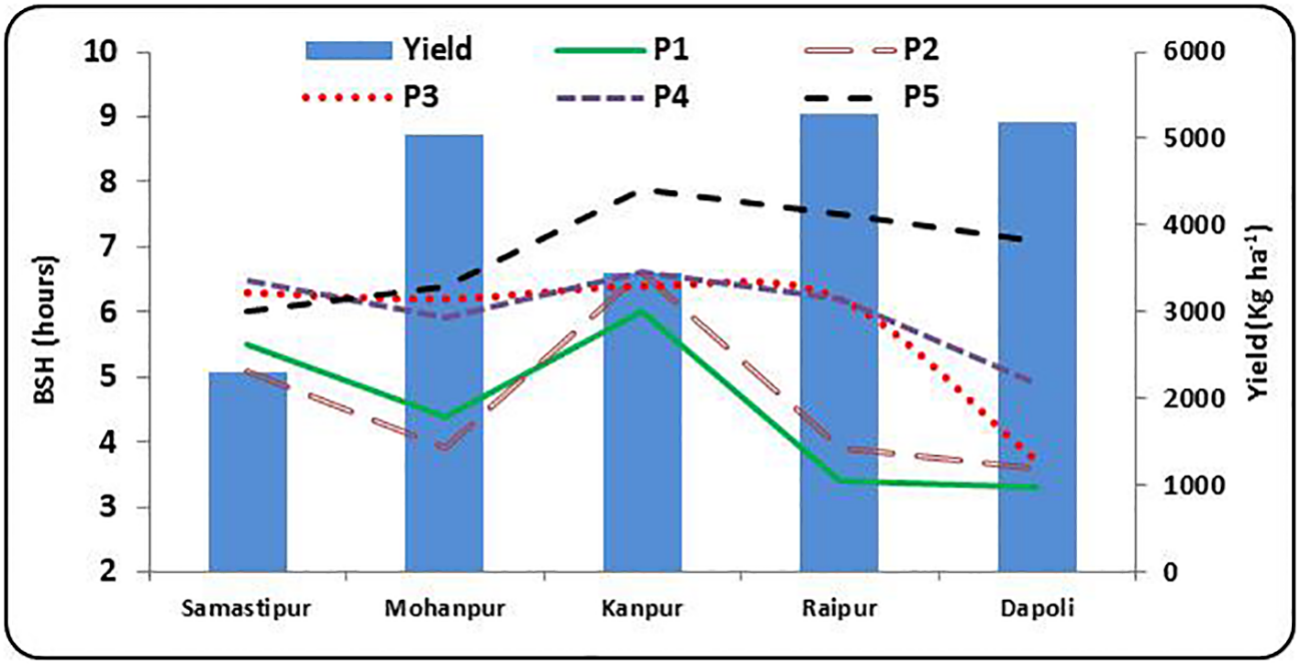
Bright sunshine hours (BSH) at growth stages vis-a-vis rice yield at different rice growing ecologies of India, (P1: Sowing to Tillering, P2: Tillering to Panicle initiation, P3: Panicle initiation to flowering, P4: Flowering to milk; P5: Milk to Physiological maturity).

The requirements of BSH for P1 to P5 are presented in **Supplementary Table 5** and graphically in **Figure 6**. The BSH for above average yield was ≥ 6.9 hours during flowering to physiological maturity and less than 5.6 and 3.6 hours during P4 and P5 stages, respectively at Samastipur. At Mohanpur, a BSH greater than 7.4 hours and less than 5 hours during P3 to P5 contributed to above average and below average yields, respectively. Similar result was observed at Kanpur, where a BSH more than 9.0 hours and less than 4 hours during P4 and P5 contributed to above average and below average yields, respectively. In Raipur and Dapoli, above average yield years had consistently higher BSH compared to below average yield years during all the stages. However, in Dapoli, the BSH ranged uniformly from 5.3 to 7.4 hours and 2.1 to 3.8 hours for above average and below average years, respectively.

#### 3.3.5 Relative humidity (RH-1 and RH-2)

RH-1 of greater than 95 per cent contributed to above average yield, while less than 85 per cent was associated with below average yield during reproductive phase (P3-P5) at Samastipur (**Supplementary Table 6**). Similar result was observed in Dapoli with more than 95 per cent and less than 91 per cent RH-1 contributing to above and below average yields, respectively. In Mohanpur, more than 95 per cent and less than 57 per cent RH-1 during flowering to physiological maturity (P4 and P5) stages was associated with above and below average yields, respectively. At the same time, in Kanpur, while more than 95 per cent (P3 and P4) and more than 85 per cent (P5) RH-1 contributed to above average yields; an RH-1 less than 89 per cent (P3 and P4) and less than 40 per cent (P5) contributed to below average yields. Same pattern was observed in Raipur with more than 92 per cent (P3 and P4) and 88 per cent (P5) contributing to above average; 45-60 per cent during P3-P4 and less than 83 per cent during P5 contributing to below average yields.

RH-2 during tillering to physiological maturity (P2-P5) was in the range of 40-70 per cent at Mohanpur and 40-85 per cent at Dapoli during above average yield years. At the same time, a higher RH-2 in the range of 78-92 per cent at Mohanpur and 92-95 per cent at Dapoli was associated with below average yield years. The reverse was observed for Samastipur and Kanpur, where, RH-2 in the range 81-98 per cent and 75-83 per cent, respectively contributed to above average yields. The below average yield years was associated with an RH-2 value of 37-50 per cent at Samastipur and 35-41 per cent at Kanpur, respectively. At Raipur, RH-2 greater than 85 per cent and less than 44 per cent during panicle initiation to milk stages contributed above and below average yields, respectively.

### 3.4 Effect of sowing environment on rice phenophase

**Figure 7:**
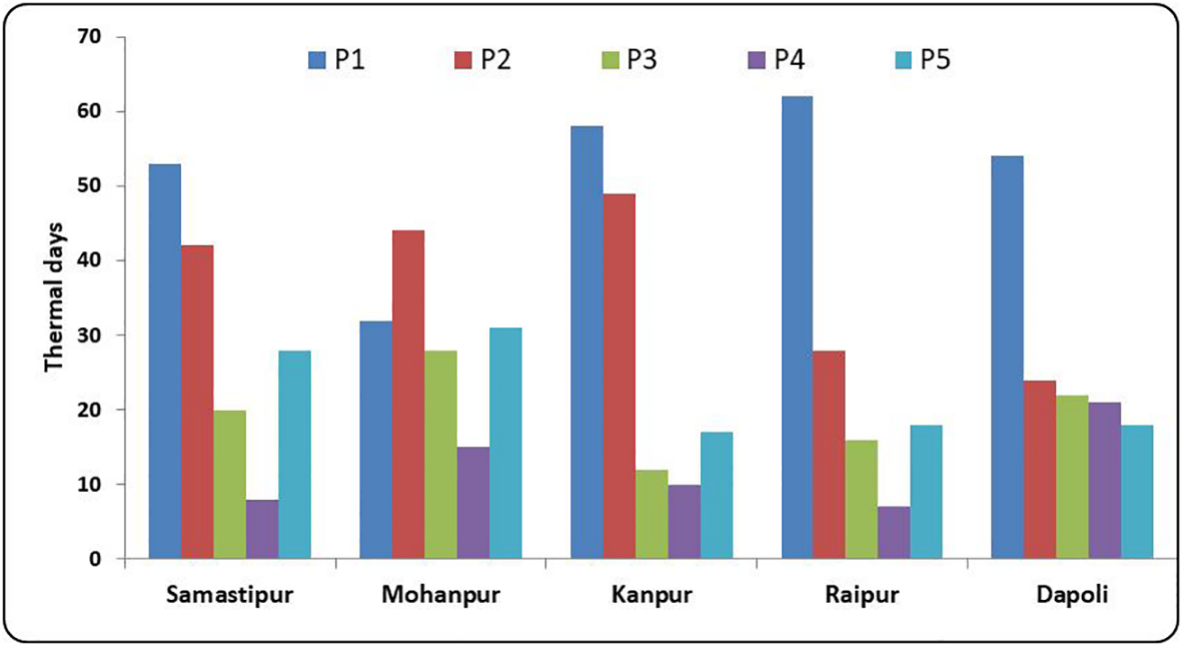
Thermal time (days) required for completion of growth stages of rice at different rice growing ecologies of India, (P1: Sowing to Tillering, P2: Tillering to Panicle initiation, P3: Panicle initiation to flowering, P4: Flowering to milk; P5: Milk to Physiological maturity).

There existed a large variation in the number of days taken for different phenological stages across different locations (**Figure 7**). The lowest number of days taken for sowing to tillering (P1) was recorded at Mohanpur (32 days), while the highest was at Raipur (62 days). Higher temperatures during the early growth phases of rice at Mohanpur might have contributed to faster vegetative growth from sowing to tillering. Islam et al. (2019) reported distinct variations in the duration of phenological stages mainly due to the thermal environment of the crop. The rate of tillering in rice tends to increase under an elevated temperature regime. The effect of temperature on tillering is affected by the duration of sunlight (Mahbubul et al., 1985). The thickness of the bran was higher under high temperatures during tillering stage (Tashiro and Wardlaw, 1989). Over Indo-Gangetic plains (Mohanpur, Samastipur, and Kanpur), the days taken for P2 stage varied from 42 to 49 days. The lowest number of days (24 days) for the P2 stage was recorded at Dapoli. The lowest (12) and the highest (28) days for the P3 stage were associated with Kanpur and Mohanpur, respectively. A small variation in the number of thermal days for the P4 stage was recorded across different locations, except at Dapoli and Mohanpur. P5 stage recorded the highest duration of 31 days at Mohanpur and the lowest (17 days) at Kanpur. The duration of various phenophases depended on temperature variations during different phonological stages and growing degree days. Alvarado (2002) reported linear relation between days taken from sowing to flowering in rice with average air temperature.

### 3.5 Phasic contribution of weather variables on yield

**Figure 8:**
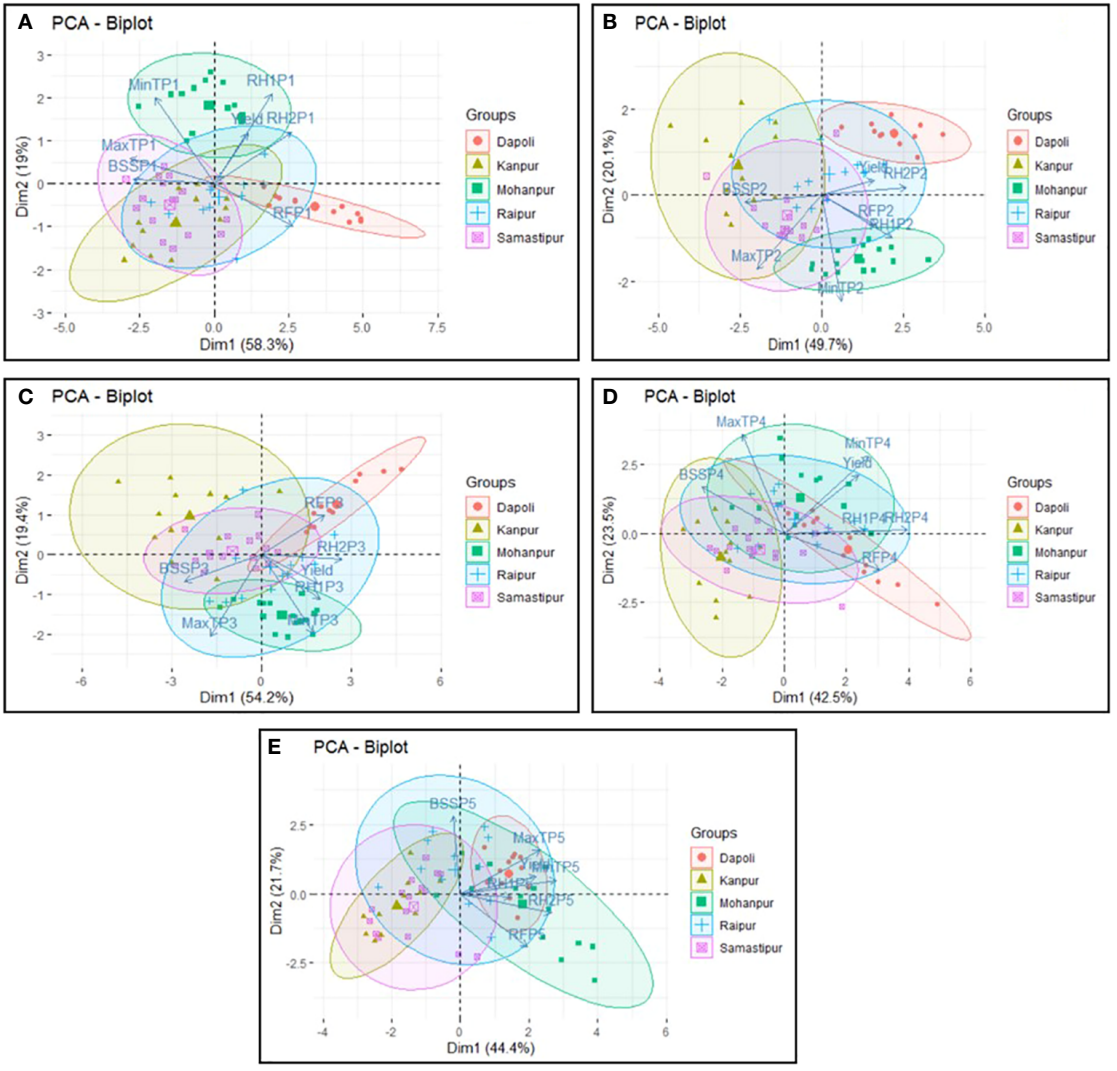
Biplot of first two principal components during **(A)** sowing to tillering **(B)** tillering to panicle initiation **(C)** panicle initiation to flowering **(D)** flowering to milk and **(E)** milk to physiological maturity.

The Principal Component Analysis (PCA) was performed and the first two principal components (PC1 and PC2) explained major yield variability across the locations. These two PCs during P1, P2, P3, P4 and P5 could explain 77 per cent, 70 per cent, 74 per cent, 66 per cent and 66 per cent of the yield variability, respectively (**Figures 8A–E**). In the biplots, two vectors with an angle less than 90 degrees are positively correlated and two vectors with an angle greater than 90 degrees are not correlated. An angle close to 180 degrees indicates the variables are negatively correlated. During P1 in Samastipur, majority of the points are lying in the third and fourth quadrants. It revealed that increase in Tmax and BSH led to decrease in the yield (**Figure 8A**). The location in the fourth quadrant i.e., Dapoli, was characterized by higher and more variable rainfall, as indicated by the length of the vector.

Mohanpur, in second and first quadrants were characterized by Tmin, RH-1 and RH-2. Humidity levels were very closely aligned to yield, thus indicating high positive correlation. In the locations from the second and third quadrants (Samastipur & Raipur), Tmax and BSH were observed to be playing crucial role. During tillering to panicle initiation (P2), yield was closely aligned with PC1; and humidity level and rainfall were observed to have positive correlation with yield at Samastipur and Raipur (**Figure 8B**). Increase in Tmax and longer duration of BSH during this stage had detrimental impact on yield at Samastipur, Kanpur and Raipur. During panicle initiation to flowering (P3), increase in RH-1 and RH-2 along with increase in Tmin were associated with high yield at Raipur and Samastipur (**Figure 8C**). However, increase in BSH had negative correlation with yield at these locations. Dapoli was characterized by higher rainfall during P3 stage, leading to higher yield. Increase in Tmin followed by increase in humidity level during flowering to milk (P4) stage, at all the locations, except Kanpur had positive effect on yield (**Figure 8D**). During milk to physiological maturity (P5), higher yield associated with increase in T_max_ and T_min_, humidity level and rainfall at all the locations, except Kanpur (**Figure 8E**).

## 4 Discussion

### 4.1 Rice yield influenced by weather elements

#### 4.1.1 Maximum temperature (T_max_) and bright sunshine hour

Due to the varying sensitivity of different rice ecosystems under study to temperature, the effect of air temperature on rice growth would be site-specific. Though average yield at Dapoli and Raipur was comparable, the above average yield at Dapoli was higher than Raipur. This may be attributed to higher maximum temperature (a difference of 5-8 °C) during reproductive stages combined with higher T_min_ (2-7 °C). It was also found that across all locations, the above average yield could be associated with higher range of T_max_ compared to that of below average yield. This result is in contrary to results reported by most of researchers (Satake and Yoshida, 1978; Yin et al., 1996; Matsui et al., 1997; Farrell et al., 2006). Therefore, the above average yield may be attributed to higher BSH during reproductive stage of above average yield years.

A considerable difference between BSH during reproductive stages of above average and below average yield years (ranging 2.8 to 7.8 hours during P5 and 1.7 to 5.1 during P4 stages) could have contributed to the higher yields. The difference in rice yield between the growing environments in the tropics is due to the difference in solar radiation between them, especially during the ripening phase (Yoshida, 1981; Yang et al., 2008). It is generally believed that higher grain yield is associated with higher incident solar radiation and higher intercepted solar radiation by rice crop canopy (Katsura et al., 2008).

#### 4.1.2 Minimum temperature (T_min_)

Across the locations, no clear-cut pattern could be identified to establish the impact of T*min* on the rice yield. However, in Mohanpur, a sudden increase in T_min_ by 8-10 °C (> 30 °C) during reproductive period compared to above average yield years resulted in 40-50 per cent reduction in yield. The reduction in yield during reproductive stage due to increased maximum as well as minimum temperatures may be due to early grain filling, formation of chalky grains and forced maturity which led to reduction in grain size and subsequently yield (Farooq et al., 2011; Krishnan et al., 2011; Lobell et al., 2012; Rahman et al., 2017). It was reported 10 per cent reduction in rice yield for 1 °C increase in minimum temperature in the dry season, though the effect of maximum temperature on crop yield was not significant (Peng et al., 2004). Whereas in Dapoli, the yield reduction was attributed to a sudden decrease in T_min_ (< 20 °C) during milk to physiological maturity. Low temperature reduced the rate of grain and dry matter formation, extends the grain filling duration and delays grain maturation (Sridevi and Chellamuthu, 2015). Night temperature of less than 19 °C is the critical low temperature for inducing grain sterility in rice (Abeysiriwardena et al., 2002).

#### 4.1.3 Rainfall

Since rice is grown under rainfed crop in India with receipt of monsoon rainfall, poor and erratic monsoon rainfall seriously interferes with crop growth and yield. One critical reason for the highest rice yield at Dapoli across the five locations may due to minimal rainfall (< 30 mm), during milk to physiological maturity stage (P5), in addition to other weather factors. The results indicated that a drier period (without rainfall) during milk to physiological maturity is desirable for higher rice productivity.

#### 4.1.4 Relative humidity

In costal locations (Dapoli and Mohanpur), an increase in RH-2 during reproductive stages significantly reduced the yield. Excess relative humidity could have led to reduced Spikelet fertility (Sridevi and Chellamuthu, 2015) besides increase in biotic stresses like pest and disease infestation. Higher relative humidity at the flowering stage under increased temperature negatively affects spikelet fertility (Yan et al., 2010). A relative humidity of 85-90 per cent at the heading stage induces almost complete grain sterility in rice at a day/night temperature of 35/30 °C (Abeysiriwardena et al., 2002). Weerakoon et al. (2008) reported that spikelet fertility was not always inhibited by high humidity, because at low temperature, fertility was high. Both higher maximum and higher minimum temperatures with high relative humidity decrease rice yields due to spikelet sterility (Peng et al., 2004). Combined effect of temperature and relative humidity is a predominant controlling factor in rice cultivation due to their spatial and temporal variability. Whereas in inland locations (Samastipur, Kanpur), a decrease in RH-2 during both vegetative and reproductive stages (25-40 per cent reduction), decreased the yield drastically. This may be attributed to higher thermal load leading to water related stresses resulting in yield loss. Whereas, the higher the relative humidity during morning hours, the more was the yield. This was particularly true during panicle initiation to physiological maturity stages. Though we have tried to describe the yield variability to weather parameters across the locations, it is to be noted that the loses of rice yields are not only caused by weather parameters, but due to other disasters also (Lesk et al., 2016).

### 4.2 Comparison of rice yield influenced by weather elements across locations

The below average yield of Mohanpur (3459 kg ha^-1^), which was on par with the above average yield of Samastipur (3517 kg ha^-1^), may be probably due to prevalence of relatively favorable temperature during critical (flowering to physiological maturity) growth stages at Mohanpur. A decrease in Tmax by 2.2 - 6.9 °C at Samastipur, compared to Mohanpur during reproductive to physiological maturity stages might have affected seed setting and formation of chaffy grains in rice. Temperature below optimum level induces high sterility in rice (Ghosh et al., 1983).

The average yield at Raipur was much higher (> 1800 kg ha^-1^) compared to Mohanpur, Samastipur and Kanpur. Though the T_max_, rainfall and BSH during P4-P5 stages were comparable at all the locations, T_min_ at Kanpur remained below 20 °C consistently. Being located in northern India in upper Gangetic plains, perhaps early onset of low temperature during reproductive to maturity phases close to the start of winter season played adverse effect on grain yield. Abeysiriwardena et al. (2002) also reported that in Sri Lanka night temperature of less than 19 °C is the critical low temperature for inducing grain sterility in rice.

While comparing the performance of rice, we could observe a specific pattern among coastal versus inland locations. At both the coastal locations, the rainfall received during P5 stage was very less (< 60 mm and < 160 mm, respectively) contributing comparatively higher yields (6650 and 6624 kg ha^-1^, respectively for Dapoli and Mohanpur). In land locations viz., Samastipur and Raipur received higher rainfall (> 325 mm) during milk to physiological maturity. While comparing above and below average yields across locations, we found that both in Dapoli and Mohanpur (Costal locations), a higher T_max_ (36-43 °C) during reproductive stage (P3, P4 and P5 phenophases) contributed to above average yield, compared to lower T_max_ (27-31 °C) during below average yield years. Although high temperature may not be conducive during flowering, however it tends to favor grain development during maturity phase. The analysis suggests that higher level of relative humidity in association with T_max_ above 40 °C during reproductive phase (P3, P4 and P5 phenophases) have beneficial impact on rice yield in Dapoli. We could not find any previous reports with similar results.

Principal component analysis (PCA) provided a comprehensive overview of rice yield in response to different climatic parameters across different rice ecologies. The score plot of PCA highlighted crucial information on yield in relation to the growing season expressed in terms of phonological phases. For instance, at Dapoli, rainfall was observed to be the crucial factor affecting yield, it was positively associated with yield at first and fourth stage and negatively associated at the final stage. Similarly, high maximum temperature and long sunshine hours were observed to have detrimental impact on yield at Kanpur. The results of the PCA offered a more in-depth approach to reveal the effects of different weather factors at different phonological phases on the yield of rice grown across different ecologies. It can serve as a guide for devising customized management practices suitable for specific ecology. Ahmed et al. (2019) used PCA to identify the climate factors affecting wheat growth and productivity.

Same rice cultivar Swarna performed differently during different sowing dates in a location as well as across the locations, which was evident from the range of yield (6650 kg ha^-1^ at Dapoli to 1101 kg ha^-1^ at Samastipur). At coastal locations like Dapoli and Mohanpur, the seasonal rainfall was much higher compared to other inland locations. In addition, the longer duration of days between panicle initiation to physiological maturity at Dapoli and Mohanpur might have contributed to higher yield. Similar result was reported by Vergara et al. (1966). Another reason could be, Swarna being a long duration variety, the variability in weather elements across the locations and between the different sowing dates within a location might had a greater influence on growth and yield. Apart from weather parameters, other factors namely soil fertility, biotic stresses, crop management practices contributed to the yield variability. Biotic stresses include insect pests, fungus, bacteria, viruses, and herbicide toxicity. Abiotic stresses include such as drought, high salinity, high or low temperatures, flooding, high light, ozone, low nutrient availability, mineral deficiency, heavy metals, pollutants, wind, and mechanical injury (Anami et al., 2020). It is generally believed that all these stresses are considered as a serious threat to sustainable paddy production. In a single location, the major reason for yield variability was variable sowing time, by which crop were exposed to different growing environments.

## 5 Conclusions

The field experiments were conducted over five locations representing different rice ecologies involving long duration rice cultivar Swarna for identifying weather thresholds to maintain sustainable rice production. The study could identify the impact of individual as well as combined effect of weather elements on rice yields. The influence of coastal and inland weather on rice yields was also quantified. It was found that across all locations, the above average yield could be associated with higher range of maximum temperature compared to that of below average yield. A relatively dry period during milk to physiological maturity was found to be desirable for higher rice productivity. A considerable difference between BSH during reproductive stages of above average and below average yield years might have contributed to the higher yields. We could find that the yield potential of the variety Swarna could be harnessed in coastal compared to inland areas. However, we should keep in mind that the individual effects of the weather variables on rice yield are difficult to distinguish because they are not often independent. We hope that the location-specific thresholds of weather parameters identified in this study will support climate resilient rice production.

## Data availability statement

The original contributions presented in the study are included in the article/**Supplementary Material**. Further inquiries can be directed to the corresponding authors.

## Author contributions

Conceptualization: SKB and AS. Methodology: SKB, AS, SA. Investigation: AS, JL, SB, VM, CS. Data Curation: AR, N. Writing—Original Draft: AS, SKB, SA. Writing—Review and Editing: AR, NM, SS, VS. Visualization: AS, NM. All authors contributed to the article and approved the submitted version.
