# Supplementary material for: Critical weather limits for paddy rice under diverse ecosystems of India
Source: Front Plant Sci. 2023 Aug 9;14:1226064. doi: 10.3389/fpls.2023.1226064 (PMC10445142; doi:10.3389/fpls.2023.1226064)
Supplement: Supplementary file 1 [file DataSheet_1.pdf]

Supplementary Material

Critical weather limits for paddy rice under diverse ecosystems of India

Santanu Kumar Bal[1], Abdus Sattar[2]*, Nidhi[3], Sarath Chandran M. Alickal [1]*, Abburi Venkata M. Subba Rao[1], Narayanan Manikandan[1], Saon Banerjee[4], Jawahar L. Choudhary[5], Vijay G. More[6], Chandra B. Singh[7], Sandeep S. Sandhu[8], Vinod Kumar Singh[1]

[1]ICAR-Central Research Institute for Dryland Agriculture, Santoshnagar, Hyderabad, India

[2&3] Dr. Rajendra Prasad Central Agricultural University, Pusa, Samastipur, Bihar, India

[4] Department of Agricultural Meteorology and Physics, BCKV, Mohanpur, West Bengal, India

[5] Department of Agrometeorology, IGKV, Raipur, Chhattisgarh, India

[6] Dr. Balasaheb Sawant Konkan Krishi Vidyapeeth, Dapoli, Maharashtra, India

[7] Chandrashekhar Azad University of Agriculture and Technology, Kanpur, Uttar Pradesh, India

[8] Department of Agricultural Meteorology and Climate Change, PAU, Ludhiana, Punjab, India

*** Correspondence:**

Abdus Sattar
sattar.met@gmail.com

Sarath Chandran M.A.
sarathagri@gmail.com

1 Supplementary Data

Supplementary Table 1

Simple linear regression models using weather parameters for different phenological stages of Rice (cultivar Swarna) at different locations in India

A. Eastern India

1. Samastipur

T_{max} (°C)						
	Statistics	P1	P2	P3	P4	P5
	intercept	14044.53	20337.07	-21909.6	-7865.9	12418.43
	Linear	-355.49	-549.539	737.8	325.4	254.12

	t-value	-1.075	-2.148	3.769	4.149	-1.663
	Pr > t	NS	0.05	0.002	0.001	0.048
	Adj. R2	0.113	0.194	0.468	0.519	0.21
	Pr > F	NS	0.05	0.002	0.001	0.048
T_{min} (˚C)						
		P1	P2	P3	P4	P5
	intercept	5363.82	-5323.36	-13773.2	2983.77	2615.66
	Linear	-119.43	304.98	691.88	-32.04	-17.79
	t-value	-0.362	0.873	4.481	-0.228	-0.127
	Pr > t	NS	NS	0.001	NS	NS
	Adj. R2	-0.062	-0.016	0.56	-0.0667	-0.07
	Pr > F	0.723	NS	0.001	NS	NS
BSH (hours)						
		P1	P2	P3	P4	P5
	intercept	-733.42	3614.08	-7370.65	-715.17	1112.41
	Linear	554.38	-258.16	1500.68	502.94	215.23
	t-value	1.676	-0.644	4.611	2.663	0.432
	Pr > t	NS	NS	0	0.019	0.018
	Adj. R2	0.108	-0.041	0.575	0.289	0.194
	Pr > F	NS	NS	0	0.019	0.018
Rainfall (mm)						
		P1	P2	P3	P4	P5
	intercept	1541.12	3641.23	1887.29	2714.89	2219.56
	Linear	3.274	-3.14	3.92	1.814	3.97
	t-value	1.158	-1.245	1.596	0.671	0.728
	Pr > t	0.042	0.037	NS	0.008	NS
	Adj. R2	0.313	0.221	0.094	0.207	-0.032
	Pr > F	0.281	NS	NS	NS	NS
RH-1 (m) (per cent)						
		P1	P2	P3	P4	P5
	intercept	12296.38	7458.75	-22037.5	-8513.23	-12359
	Linear	-108.49	-57.22	270.33	120.66	164.58

	t-value	-2.964	-0.194	1.128	1.038	1.076
	Pr > t	0.01	NS	NS	NS	NS
	Adj. R2	0.342	-0.069		0.005	0.01
	Pr > F	0.01	NS	NS	NS	NS
RH-2 (e) (per cent)						
		P1	P2	P3	P4	P5
	intercept	794.71	-342.67	-1729.74	1870.71	-384.59
	Linear	21.63	38.39	64.23	7.71	55.25
	t-value	0.231	0.604	1.623	0.259	0.913
	Pr > t	NS	NS	NS	NS	NS
	Adj. R2	-0.067	-0.044	0.098	-0.066	-0.011
	Pr > F	0.82	NS	NS	NS	NS

P1: Sowing to Tillering, P2: Tillering to Panicle initiation, P3: Panicle initiation to flowering, P4: Flowering to milk; P5: Milk to Physiological maturity

2. Mohanpur

T_{max} (°C)						
		P1	P2	P3	P4	P5
	intercept	-16192.5	-3709.54	8243.21	-1742.26	2317.83
	Linear	639.226	265.293	-73.241	187.141	101.32
	t-value	2.164	0.47	-0.112	0.833	0.285
	Pr > t	0.048	NS	0.045	0.031	0.009
	Adj. R2	0.197	-0.055	0.23	0.271	0.312
	Pr > F	0.048	NS	0.045	0.031	0.009
T_{min} (°C)						
		P1	P2	P3	P4	P5
	intercept	-34313.8	-10890.5	-3245.49	-4237.22	-381.914
	Linear	1496.77	604.643	412.247	447.215	318.223
	t-value	2.015	0.417	1.234	2.171	2.014
	Pr > t	NS	NS	0.004	0.01	0.024
	Adj. R2	0.169	-0.058	0.272	0.284	0.301
	Pr > F	NS	NS	0.004	0.01	0.024

BSH (hours)						
		P1	P2	P3	P4	P5
	intercept	3598.33	6083.387	-2211.12	-3166.9	-2270.74
	Linear	331.36	567.369	1148.98	1332.05	1141.47
	t-value	1.162	-0.426	6.37	7.048	5.853
	Pr > t	NS	NS	0.000	0.041	0.047
	Adj. R2	0.023	-0.058	0.725	0.764	0.689
	Pr > F	0.265	0.676	0.000	0.041	0.047
Rainfall (mm)						
		P1	P2	P3	P4	P5
	intercept	4512.712	-354.5	4569.981	4914.221	7623.65
	Linear	-2.145	9.264	3.11	13.247	-17.9
	t-value	-2.871	1.247	0.704	2.437	-1.677
	Pr > t	0.005	0.045	0.493	0.041	0.036
	Adj. R2	0.291	-0.214	-0.035	0.312	0.108
	Pr > F	0.005	0.045	0.493	0.041	0.036
RH-1 (m) (per cent)						
		P1	P2	P3	P4	P5
	intercept	47645.54	-19966.5	-56.313	7680.32	6019.905
	Linear	-445.238	261.014	53.806	-28.621	-10.66
	t-value	-2.284	0.696	0.341	-0.381	-0.132
	Pr > t	0.039	NS	NS	NS	NS
	Adj. R2	0.219	-0.036	-0.063	-0.06	-0.07
	Pr > F	0.039	0.498	0.738	0.709	0.897
RH-2 (e) (per cent)						
		P1	P2	P3	P4	P5
	intercept	16604.57	-7366.95	-1503.79	342.419	-157.346
	Linear	-141.117	156.085	88.679	70.601	82.445
	t-value	-2.158	1.419	1.062	1.649	2.151
	Pr > t	0.049	NS	NS	NS	0.049
	Adj. R2	0.196	0.063	0.009	0.103	0.195
	Pr > F	0.049	0.178	0.306	0.121	0.049

P1: Sowing to Tillering, P2: Tillering to Panicle initiation, P3: Panicle initiation to flowering, P4: Flowering to milk; P5: Milk to Physiological maturity

B. Northern India

Kanpur

T_{max} (°C)						
		P1	P2	P3	P4	P5
	intercept	3944.37	4092.743	1677.411	-5306.3	-6515.03
	Linear	-7.001	-11.476	62.206	284.734	337.391
	t-value	-0.034	-0.051	0.5	2.997	1.564
	Pr > t	NS	NS	NS	0.013	NS
	Adj. R2	-0.1	-0.1	-0.073	0.421	0.116
	Pr > F	0.974	0.96	0.628	0.013	0.149
T_{min} (°C)						
		P1	P2	P3	P4	P5
	intercept	1043.33	-142.769	104.949	-334.683	1981.012
	Linear	107.227	163.395	190.298	252.531	126.282
	t-value	0.378	1.062	1.892	2.789	0.811
	Pr > t	NS	NS	NS	0.019	NS
	Adj. R2	-0.084	0.011	0.19	0.381	-0.032
	Pr > F	0.713	0.313	0.088	0.019	0.436
BSH (hours)						
		P1	P2	P3	P4	P5
	intercept	4755.22	10223.22	-790.21	787.76	1904.083
	Linear	-214.47	-1019.34	653.56	406.991	196.324
	t-value	-0.614	-2.949	3.704	2.579	1.508
	Pr > t	NS	0.015	0.025	0.027	NS
	Adj. R2	-0.06	0.412	0.536	0.339	0.104
	Pr > F	0.553	0.015	0.025	0.027	0.162
Rainfall (mm)						
		P1	P2	P3	P4	P5
	intercept	3076.41	1495.72	-16273.3	3182.215	3011.5

	Linear	1.651	9.259	222.12	-49.722	-14.245
	t-value	1.142	2.341	4.302	-2.473	-2.457
	Pr > t	NS	0.039	0.048	0.031	0.037
	Adj. R2	0.027	0.295	-0.024	0.337	0.412
	Pr > F	NS	0.039	NS	0.031	0.037
RH-1 (m) (per cent)						
		P1	P2	P3	P4	P5
	intercept	4631.32	689.698	-9907.26	-16273.3	1352.946
	Linear	-10.789	35.266	158.62	222.15	27.199
	t-value	-0.178	0.272	4.655	4.302	0.356
	Pr > t	NS	NS	0.001	0.024	NS
	Adj. R2	-0.097	-0.092	0.653	0.614	-0.086
	Pr > F	NS	0.791	0.001	0.024	0.729
RH-2 (e) (per cent)						
		P1	P2	P3	P4	P5
	intercept	2770.68	697.707	1887.204	3504.106	3092.771
	Linear	13.45	45.667	34.625	4.488	13.981
	t-value	0.399	0.809	1.53	0.133	0.319
	Pr > t	NS	NS	NS	NS	NS
	Adj. R2	-0.083	-0.032	0.109	-0.098	-0.089
	Pr > F	NS	0.437	0.157	0.897	0.756

P1: Sowing to Tillering, P2: Tillering to Panicle initiation, P3: Panicle initiation to flowering, P4: Flowering to milk; P5: Milk to Physiological maturity

C. Central India

1. Raipur

T_{max} (°C)						
		P1	P2	P3	P4	P5
	intercept	2315.17	4954.193	-4445.15	4915.01	2974.38
	Linear	81.25	-4.379	303.61	-1.874	67.19
	t-value	0.715	0.084	2.284	-0.009	0.614

	Pr > t	0.05	0.009	0.045	0.001	0.007
	Adj. R2	0.213	0.224	0.277	0.372	0.342
	Pr > F	0.05	0.009	0.045	0.001	0.007
T_{min} (°C)						
		P1	P2	P3	P4	P5
	intercept	-4924.19	1603.458	-15329.3	-2264.16	3353.368
	Linear	407.94	148.67	843.53	321.255	95.341
	t-value	1.107	0.324	2.959	3.309	0.805
	Pr > t	NS	NS	0.014	0.008	NS
	Adj. R2	0.02	-0.089	414	0.475	-0.033
	Pr > F	NS	0.753	0.014	0.008	0.439
BSH (hours)						
		P1	P2	P3	P4	P5
	intercept	4195.26	2101.65	2783.48	3465.31	3933.759
	Linear	323.77	796.59	360.36	294.38	180.968
	t-value	1.021	3.777	3.51	2.391	1.095
	Pr > t	NS	0.004	0.023	0.038	NS
	Adj. R2	0.004	0.547	0.507	0.300	0.018
	Pr > F	NS	0.004	0.023	0.038	NS
Rainfall(mm)						
		P1	P2	P3	P4	P5
	intercept	5847.21	5314.27	4970.009	5032.355	5319.054
	Linear	-0.89	-1.211	2.517	6.228	-1.8
	t-value	-1.234	-1.651	1.594	2.377	-0.177
	Pr > t	0.047	0.036	NS	0.039	NS
	Adj. R2	0.221	0.354	0.123	0.297	-0.097
	Pr > F	0.047	0.036	0.142	0.039	0.863
RH-1 (m) (per cent)						
		P1	P2	P3	P4	P5
	intercept	7122.82	6983.084	3229.222	3880.153	4675.242
	Linear	-20.79	-18.73	22.616	15.624	6.996
	t-value	-0.45	-0.474	0.606	0.69	0.398

	Pr > t	NS	0.646	0.558	0.506	0.699
	Adj. R2	-0.078	-0.076	-0.061	-0.05	-0.083
	Pr > F	NS	0.646	0.558	0.506	0.699
RH-2 (e) (per cent)						
		P1	P2	P3	P4	P5
	intercept	6871.69	7666.851	3248.31	3221.603	6147.365
	Linear	-21.94	-32.736	30.595	32.372	-18.317
	t-value	-0.814	-0.715	1.199	1.97	-0.719
	Pr > t	NS	0.419	0.258	0.077	0.489
	Adj. R2	-0.032	-0.047	0.038	0.208	-0.046
	Pr > F	NS	0.491	0.258	0.077	0.489

P1: Sowing to Tillering, P2: Tillering to Panicle initiation, P3: Panicle initiation to flowering, P4: Flowering to milk; P5: Milk to Physiological maturity

D. South-Western India

Dapoli

T_{max} (˚C)						
		P1	P2	P3	P4	P5
	intercept	-15749.9	-19354.8	6899.794	1795.072	-7811.44
	Linear	733.31	859.08	-59.02	111.399	393.571
	t-value	5.383	1.65	-0.119	0.807	1.679
	Pr > t	0.000	NS	NS	NS	NS
	Adj. R2	0.718	0.135	-0.098	-0.033	0.142
	Pr > F	0	0.13	0.907	0.807	0.124
T_{min} (˚C)						
		P1	P2	P3	P4	P5
	intercept	-6327.65	-1526.78	15153.43	7015.798	3961.511
	Linear	483.27	2.83.749	-435.248	-80.39	57.142
	t-value	1.266	0.439	-0.931	-0.173	0.457
	Pr > t	NS	NS	NS	NS	NS
	Adj. R2	0.052	-0.079	-0.012	-0.097	-0.078
	Pr > F	0.234	0.67	0.374	0.866	0.658
BSH (hours)						
		P1	P2	P3	P4	P5
	intercept	4425.7	5185.275	4775.534	4769.411	4631.3
	Linear	333.09	0.279	117.514	93.194	83.245
	t-value	1.946	0.002	0.542	0.602	0.374
	Pr > t	NS	NS	0.006	0.050	0.031
	Adj. R2	0.202	-0.1	0.241	0.211	0.3
	Pr > F	NS	NS	0.006	0.051	0.031
Rainfall(mm)						
		P1	P2	P3	P4	P5
	intercept	4852.38	4712.81	4387.28	4925.23	5128.29
	Linear	-0.641	-0.189	0.631	-0.279	-13.77
	t-value	-2.013	-0.231	0.841	-0.522	-1.273

	Pr > t	0.002	0.049	0.021	0.028	0.036
	Adj. R2	0.314	0.283	0.199	0.022	0.251
	Pr > F	0.108	0.897	0.51	0.682	0.356
RH-1 (m) (per cent)						
		P1	P2	P3	P4	P5
	intercept	19956.16	-34821.5	-16058.3	4933.8	-22867.9
	Linear	-157.41	425.237	226.207	2.7	303.562
	t-value	-1.944	1.352	1.237	0.024	2.578
	Pr > t	NS	NS	NS	NS	0.028
	Adj. R2	0.202	0.07	0.046	-0.1	0.339
	Pr > F	0.081	0.206	0.244	0.981	0.028
RH-2 (e) (per cent)						
		P1	P2	P3	P4	P5
	intercept	14772.74	13458.66	11721.97	7612.02	7191.599
	Linear	-109.56	-97.418	-78.272	-30.803	-28.923
	t-value	-3.548	-1.346	-1.724	-1.128	-0.848
	Pr > t	0.005	NS	NS	NS	NS
	Adj. R2	0.513	0.069	0.152	0.024	-0.026
	Pr > F	0.005	0.208	0.115	0.286	0.417

T_{max} (˚C) is Maximum temperature, T_{min} (˚C) is minimum temperature, BSH is Bright sunshine hour, RH (m) (per cent) is Morning relative humidity, RH (e) (per cent) is evening relative humidity.

P1: Sowing to Tillering, P2: Tillering to Panicle initiation, P3: Panicle initiation to flowering, P4: Flowering to milk; P5: Milk to Physiological maturity

Supplementary Table 2

Thresholds of maximum temperature (°C) during different phenological stages of wet season rice (cultivar Swarna) at different stations in India for obtaining three different categories of yield

Yield Category	P1	P2	P3	P4	P5	Yield (Kg ha^{-1})
**Samastipur**						
Above average	29.6	30.6	34.5	34.9	24.3	3517
Average	33.0	32.8	32.8	31.3	26.7	2309
Below Average	36.4	35.0	31.2	27.6	30.1	1101
**Mohanpur**						
Above average	30.7	27.0	38.3	35.9	38.0	6624
Average	33.2	33.0	33.7	33.5	32.7	5042
Below Average	35.7	39.0	29.2	31.1	27.5	3459
**Kanpur**						
Above average	33.4	35.0	37.4	34.4	32.7	4495
Average	39.1	31.2	29.7	30.8	29.6	3458
Below Average	27.2	29.4	27.0	27.1	26.5	2421
**Raipur**						
Above average	41.3	37.0	34.4	35.9	35.3	5991
Average	31.8	31.1	32.1	31.6	31.3	5288
Below Average	29.4	34.2	29.7	29.0	24.2	4585
**Dapoli**						
Above average	34.6	33.8	43.3	40.5	43.4	6650
Average	28.6	28.6	29.0	30.4	33.0	5186
Below Average	27.6	27.7	29.3	27.0	31.2	4463

P1: Sowing to Tillering, P2: Tillering to Panicle initiation, P3: Panicle initiation to flowering, P4: Flowering to milk; P5: Milk to Physiological maturity

Supplementary Table 3

Thresholds of minimum temperature (°C) during different phenological stages rice at different stations in India for obtaining three different categories of yield

Yield Category	P1	P2	P3	P4	P5	Yield (Kg ha^{-1})
**Samastipur**						
Above average	25.5	29.0	24.9	22.2	19.7	3517
Average	25.6	25.0	23.2	21.1	20.3	2309
Below Average	35.7	21.1	21.5	22.8	23.2	1101
**Mohanpur**						
Above average	25.2	23.7	21.6	20.9	19.7	6624
Average	26.3	26.3	25.8	24.8	23.8	5042
Below Average	27.4	29.0	29.9	28.8	30.6	3459
**Kanpur**						
Above average	32.3	28.4	23.1	19.1	20.0	4495
Average	22.6	22.0	19.6	18.0	13.7	3458
Below Average	12.9	25.7	22.2	20.9	22.5	2421
**Raipur**						
Above average	26.8	29.5	25.3	25.7	27.7	5991
Average	25.0	24.8	24.4	23.5	20.3	5288
Below Average	23.3	20.0	23.6	21.3	16.9	4585
**Dapoli**						
Above average	33.1	39.4	32.6	32.8	29.6	6650
Average	23.8	23.7	22.9	22.8	21.5	5186
Below Average	22.3	21.1	24.6	31.7	18.8	4463

P1: Sowing to Tillering, P2: Tillering to Panicle initiation, P3: Panicle initiation to flowering, P4: Flowering to milk; P5: Milk to Physiological maturity

Supplementary Table 4

Thresholds of rainfall (mm) during different phenological stages of rice for obtaining three different categories of yield

Yield Category	P1	P2	P3	P4	P5	Yield (Kg ha^{-1})
**Samastipur**						
Above average	402.5	429.9	415.7	389.1	326.7	3517
Average	218.7	273.5	107.5	111.6	84.3	2309
Below Average	234.8	116.9	200.6	190.3	181.8	1101
**Mohanpur**						
Above average	630.9	753.3	357.1	106.7	156.4	6624
Average	211.7	382.5	251.8	119.5	90.2	5042
Below Average	207.5	411.6	560.6	205.7	101.8	3459
**Kanpur**						
Above average	525.4	323.9	301.7	28.3	28.8	4495
Average	224.4	211.9	229.8	61.0	31.2	3458
Below Average	385.6	99.9	121.3	24.5	38.9	2421
**Raipur**						
Above average	464.7	147.4	408.5	153.9	373.5	5991
Average	398.2	213.2	127.2	41.0	77.2	5288
Below Average	631.7	573.8	154.1	71.8	207.9	4585
**Dapoli**						
Above average	742.3	219.2	332.7	493.1	29.3	6650
Average	230.8	380.9	405.8	270.2	11.4	5186
Below Average	387.3	395.4	835.9	224.0	56.9	4463

P1: Sowing to Tillering, P2: Tillering to Panicle initiation, P3: Panicle initiation to flowering, P4: Flowering to milk; P5: Milk to Physiological maturity

Supplementary Table 5

Thresholds of bright sunshine hours during different phenological stages of rice for obtaining three different categories of yield

Yield Category	P1	P2	P3	P4	P5	Yield (Kg ha^{-1})
**Samastipur**						
Above average	7.7	3.4	3.4	7.3	6.9	3517
Average	5.5	5.1	6.3	6.9	6.0	2309
Below Average	3.3	9.7	9.1	5.6	3.6	1101
**Mohanpur**						
Above average	2.4	4.7	7.4	8.9	7.8	6624
Average	4.4	3.9	6.2	5.9	6.4	5042
Below Average	9.1	5.0	4.9	3.8	5.0	3459
**Kanpur**						
Above average	1.2	5.6	7.7	9.1	9.2	4495
Average	6.0	6.6	6.4	6.6	7.9	3458
Below Average	10.9	7.7	5.1	4.0	2.6	2421
**Raipur**						
Above average	5.5	8.4	7.7	8.5	8.9	5991
Average	3.4	3.9	6.2	6.2	7.5	5288
Below Average	1.2	3.1	4.6	3.8	3.6	4585
**Dapoli**						
Above average	5.7	5.6	5.9	7.4	5.3	6650
Average	3.3	3.6	3.7	4.9	7.1	5186
Below Average	2.1	3.4	3.8	3.6	2.1	4463

P1: Sowing to Tillering, P2: Tillering to Panicle initiation, P3: Panicle initiation to flowering, P4: Flowering to milk; P5: Milk to Physiological maturity

Supplementary Table 6

Thresholds of morning relative humidity (per cent) during different phenological stages of for obtaining three different categories of yield

Yield Category	P1	P2	P3	P4	P5	Yield (Kg ha^{-1})
**Samastipur**						
Above average	80.9	68.9	94.5	99.7	96.5	3517
Average	92.1	90.0	90.1	89.7	89.1	2309
Below Average	97.3	92.1	85.6	79.7	81.8	1101
**Mohanpur**						
Above average	99.2	89.8	65.3	97.5	95.2	6624
Average	95.7	95.8	94.8	92.2	91.7	5042
Below Average	92.1	98.1	97.2	36.9	56.7	3459
**Kanpur**						
Above average	82.6	87.8	95.1	95.9	85.5	4495
Average	80.2	78.4	91.5	90.3	77.4	3458
Below Average	74.3	49.0	81.5	88.8	39.3	2421
**Raipur**						
Above average	54.4	53.0	92.2	95.3	88.3	5991
Average	88.2	90.5	91.1	90.2	87.7	5288
Below Average	92.0	98.0	60.0	45.2	82.9	4585
**Dapoli**						
Above average	69.5	97.6	94.7	96.6	97.1	6650
Average	93.8	94.1	93.9	93.5	92.4	5186
Below Average	98.4	92.4	90.7	90.2	90.0	4463

P1: Sowing to Tillering, P2: Tillering to Panicle initiation, P3: Panicle initiation to flowering, P4: Flowering to milk; P5: Milk to Physiological maturity

Supplementary Table 7

Thresholds of afternoon relative humidity (per cent) during different phenological stages for obtaining three different categories of yield

Yield Category	P1	P2	P3	P4	P5	Yield (Kg ha^{-1})
**Samastipur**						
Above average	67.8	97.5	81.7	83.5	70.6	3517
Average	70.0	69.1	62.9	56.8	48.7	2309
Below Average	84.1	37.6	44.1	49.9	26.9	1101
**Mohanpur**						
Above average	93.2	69.4	56.0	44.1	40.1	6624
Average	82.0	79.5	73.8	66.6	59.2	5042
Below Average	70.8	89.7	91.6	89.0	78.4	3459
**Kanpur**						
Above average	87.7	83.1	75.4	80.2	80.2	4495
Average	50.9	60.4	55.4	75.2	76.1	3458
Below Average	65.9	37.7	35.4	40.7	59.0	2421
**Raipur**						
Above average	40.2	51.2	89.6	85.5	38.5	5991
Average	72.3	72.7	66.7	63.8	47.0	5288
Below Average	94.4	94.3	43.7	42.1	85.4	4585
**Dapoli**						
Above average	46.7	39.1	56.5	66.2	85.0	6650
Average	87.5	84.9	83.5	78.8	69.3	5186
Below Average	94.1	92.3	92.7	92.2	94.3	4463

P1: Sowing to Tillering, P2: Tillering to Panicle initiation, P3: Panicle initiation to flowering, P4: Flowering to milk; P5: Milk to Physiological maturity
